# Association between IL-21 gene rs907715 polymorphisms and Graves’ disease in a Southern Chinese population

**DOI:** 10.3892/etm.2014.1707

**Published:** 2014-05-12

**Authors:** HUA ZENG, HAIYAN YAN, ZHIXIAN ZHANG, WEIZHEN FANG, RUI DING, LISI HUANG, MEI CHEN, JIN ZHANG

**Affiliations:** 1 Department of Clinical Laboratory, Sun Yat-sen Memorial Hospital, Sun Yat-sen University, Guangzhou, Guangdong 510120, P.R. China; 2 Department of Endocrinology, Sun Yat-sen Memorial Hospital, Sun Yat-sen University, Guangzhou, Guangdong 510120, P.R. China

**Keywords:** interleukin-21, Graves’ disease, gene polymorphisms

## Abstract

Interleukin-21 (IL-21) is a pleiotropic cytokine linking innate and adaptive immune responses, which has been reported to play a key role in multiple autoimmune diseases. The aim of the present case-control study was to investigate the genetic association between single nucleotide polymorphisms (SNPs) of rs907715 within the IL-21 gene and Graves’ disease (GD) in a Southern Chinese population. A total of 211 patients with GD and 212 control subjects were recruited for the study. IL-21 gene rs907715 polymorphisms were detected by direct DNA sequencing. The results indicated that the frequencies of the GG genotype and the G allele in GD patients were significantly increased when compared with the frequencies in the controls (P=6.7×10^−3^ and P=2.0×10^−5^, respectively). In addition, the frequency of the AA genotype was much lower in the patient group when compared with the control group (16.6 vs. 34.0%; P=4.0×10^−5^). Furthermore, the G allele of rs907715 was associated with relapse in GD patients. These observations indicated that polymorphisms of IL-21/rs907715 may affect the susceptibility to GD in a Southern Chinese population. The G allele was significantly associated with an increased risk of GD development, whereas the A allele may lower the susceptibility to GD.

## Introduction

Graves’ disease (GD) is a common autoimmune thyroid disease accounting for ~85% of all clinical hyperthyroidism cases. Patients produce autoantibodies that activate the thyroid stimulating hormone receptor (TSHR) on thyroid follicular cells, leading to thyroid enlargement and excessive production of thyroid hormones (1–3). The etiology of GD is considered to be multifactorial, including complex interactions between environmental, genetic, endogenous and local factors (4,5). Previous studies have focused on identifying a number of putative susceptibility genes in GD and several gene loci have been reported to be associated with a risk of developing GD, including TSHR, interleukin-21 (IL-21), human leukocyte antigen class I and II, protein tyrosine phosphatase non-receptor 22 and cytotoxic T-lymphocyte associated 4 (6–13).

IL-21 is a pleiotropic cytokine that is produced mainly by activated CD4^+^ T cells and natural killer (NK) T cells (14). The activity of IL-21 is mediated by binding to a composite receptor consisting of a private receptor (IL-21R) and the common cytokine receptor γ chain (γ_c_) (15,16). The expression of IL-21R has been detected in a variety of lymphohematopoietic cells, as well as fibroblasts, keratinocytes and intestinal epithelial cells (17–19), indicating that IL-21 is involved in a wide range of biological functions. Previously, IL-21 has been shown to play a critical role in the interactions among B cells, T cells, NK cells and dendritic cells, and may serve as a pivotal cytokine linking innate and adaptive immune responses (20). According to the cellular context of costimulation and the ambient cytokine environment, IL-21 may promote the proliferation and differentiation of T cells, alter the production of cytokines and chemokines, induce the maturation and activation of NK cells and enhance antibody-dependent cellular cytotoxicity by NK cells (21–23). In addition, IL-21 has essential non-redundant regulatory functions on B cell responses and can promote B cell differentiation and antibody production (24–26), indicating that IL-21 may also be a causative factor in autoimmune diseases. IL-21 gene polymorphisms have been reported to be associated with an increasing number of autoimmune or immunological diseases, including systemic lupus erythematosus (SLE) (27), rheumatoid arthritis (28,29), GD (8) and type 1 diabetes (30).

A previous study demonstrated that GD patients from Southern China had increased serum expression levels of IL-21 (31). However, whether single nucleotide polymorphisms (SNPs) of the IL-21 gene are associated with GD susceptibility remains unclear. In the current study, rs907715 within the IL-21 gene intronic region was selected as the tag-SNP and distributions of IL-21/rs907715 gene polymorphisms among GD patients and healthy controls were analyzed. In addition, correlations among genotypes and clinical manifestations of GD were investigated. The aim of the study was to identify the association between different genotypes at the IL-21 rs907715 gene locus with GD in a Southern Chinese population.

## Materials and methods

### Subjects

For the case-control cohort, 211 GD patients (male, 70; female, 141; age, 26–46 years; mean age, 37 years) from Southern China were recruited from the Department of Endocrinology at Sun Yat-sen Memorial Hospital (Guangzhou, China). The GD patients were defined by clinical manifestations and biochemical criteria of thyrotoxicosis, including thyroid stimulating hormone levels of <0.05 mIU/l, free triiodothyronine levels of >6.5 pmol/l (normal range, 3.5–6.5 pmol/l), free thyroxine levels of >22.7 pmol/l (normal range, 11.5–22.7 pmol/l; and had positive circulating TSHR antibodies or antibodies against thyroglobulin or thyroid peroxidase (32). The levels of the hormones mentioned above were measured by ADVIA Centaur Immunoassay System (Siemens Healthcare, Erlangen, Germany).

In total, 212 control subjects (male, 78; female, 134; age, 33–50 years; mean age, 41 years) without family history of thyroid diseases or other autoimmune diseases were recruited from the Health Care Center at Sun Yat-sen Memorial Hospital. All the healthy controls were age- and gender-matched with the GD patients. Informed consent was provided by all the participants and the experimental protocol was approved by the Ethics Committee of Sun Yat-sen Memorial Hospital.

### Genotyping

**Figure 1:**
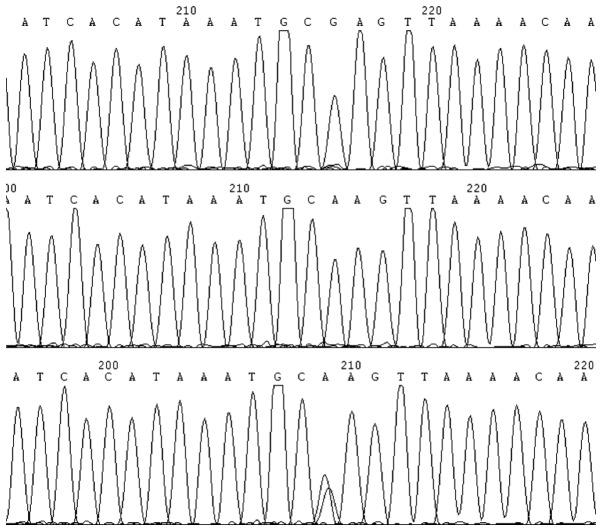
Sequencing results of the IL-21/rs907715 gene in a Southern population from China. IL, interleukin.

Genomic DNA was prepared from peripheral blood samples using a DNA extraction kit (Omega Bio-Tek, Inc., Norcross, GA, USA), according to the manufacturer’s instructions. The concentration and quality of DNA were detected by a nucleic acid/protein analyzer (Beckman Coulter, Miami, FL, USA) and agarose gel electrophoresis, respectively. SNPs of IL-21/rs907715 were determined by direct DNA sequencing following polymerase chain reaction (PCR). The forward and reverse primers for IL-21/rs907715 were 5′-CCCCAAGTTCCATAAATAGT-3′ and 5′-TTTTTGTATTTTTAGTAGAGACCA-3′, respectively. PCR was conducted using a genomic DNA template from each subject at a total volume of 50 μl, which contained 0.25 μl Ex *Taq* polymerase (5.0 U/ml; Takara Bio, Inc., Shiga, Japan), 5.0 μl 10X PCR buffer, 4.0 μl dNTP mixture, 3.0 μl DNA template (50 ng/μl), 35.75 μl PCR-grade water and 1.0 μl of each 20 μM primer. PCR conditions were as follows: 94°C for 4 min; 35 cycles of 94°C for 45 sec, 58°C for 45 sec and 72°C for 45 sec; and a final extension at 72°C for 4 min. Following amplification, the PCR products were submitted for DNA sequencing. The sequences are shown in Fig. 1.

### Clinical phenotype correlations

Correlation analyses between the genotypes/alleles of IL-21/rs907715 and the clinical characteristics of GD were performed. The detailed contents were as follows: i) Age of onset (≤30 vs. ≥31 years), the former represented early onset of GD and the latter represented the normal age of onset; ii) thyroid size (≤I vs. ≥II°), goiter size was divided into three degrees by physical examination (33); iii) presence or absence of family history of autoimmune thyroid diseases, including first-degree relatives (parents, children and siblings) and second-degree relatives (grandparents, uncles and aunts); iv) presence or absence of ophthalmopathy, which was defined as a distinctive disorder featured by inflammation and swelling of the extraocular muscles and eyelid retraction, periorbital edema, episcleral vascular injection, conjunctive swelling and proptosis (33,34); and v) presence or absence of relapse history of GD patients.

### Statistical analysis

All genotyping results were analyzed by the Hardy-Weinberg equilibrium using Excel (Microsoft Office; Microsoft, Redmond, WA). Allele and genotype frequencies between the case and control groups were compared using the χ^2^ test or Fisher’s exact test. The odds ratio (OR) and 95% confidence interval (CI) were calculated to estimate the disease susceptibility of specific genotypes and alleles. Statistical analysis was performed using SPSS software version 20.0 (IBM, Armonk, NY, USA). P<0.05 was considered to indicate a statistically significant difference.

## Results

### Genotype distributions

A Hardy-Weinberg equilibrium of the genotype distributions of IL-21/rs907715 polymorphisms was exhibited in the control group (P>0.05). The allele and genotype frequencies of IL-21/rs907715 in the case and control groups are listed in Table I. The distributions of rs907715 genotypes in GD patients (GG, 35.1%; AG, 48.3%; AA, 16.6%) differed significantly from those in the healthy controls (GG, 23.0%; AG, 43.0%; AA, 34.0%; χ^2^=18.500; P=9.6×10^−5^). As shown in Table I, the frequency of the GG genotype was significantly higher in the patients as compared with the controls (P=6.7×10^−3^; OR, 1.797; 95% CI, 1.173–2.753). Conversely, the frequency of the AA genotype was markedly lower in the patient group compared with the control group (P=4.0×10^−5^; OR, 0.387; 95% CI, 0.244–0.613). No significant difference was observed in the AG genotype distribution between the patients and matched controls (P=0.263). In addition, the G allele of IL-21/rs907715 was significantly more frequent in the GD patients than in the healthy controls. The OR for carrying the G allele was 1.807 greater in the patients as compared with the controls (P=2.0×10^−5^; 95% CI, 1.376–2.374).

### Correlation analyses

Significant differences were observed when the patients with and without relapse history were compared. The frequencies of the GG genotype in the relapse and non-relapse groups were 64.7 and 32.2%, respectively (χ^2^=12.866; P=1.6×10^−3^), and the frequency of the G allele was also significantly increased in the patients with relapse history (P=1.4×10^−3^; OR, 2.628; 95% CI, 1.427–4.840). However, no associations were observed between the other clinical phenotypes and rs907715 SNPs, including age at initial diagnosis of GD, presence or absence of goiter by palpation, presence or absence of ophthalmopathy and family history (Table II).

### Allele and genotype frequencies of IL-21/rs907715 in GD patients with and without relapse history and controls

Compared with the controls, the GG genotype and G allele of IL-21/rs907715 were markedly increased in the patients with and without relapse history. As shown in Table III, the frequencies of the G allele in the relapse and control groups were 77.9 and 44.6%, respectively (P=1.0×10^−6^; OR, 4.393; 95% CI, 2.401–8.040). Similarly, the G allele exhibited an increased frequency in the non-relapse patients when compared with the controls (P=3.9×10^−4^; OR, 1.672; 95% CI, 1.257–2.222). The frequency of the AA genotype in the relapse group was much lower compared with the control group (χ^2^=25.588; P=2.8×10^−6^). A significant correlation in AA genotype distributions was also observed between the non-relapse patients and healthy controls (χ^2^=13.910; P=9.5×10^−4^).

## Discussion

IL-21 is a potent immunomodulatory four-α-helical-bundle type I cytokine that was initially identified via functional cloning as the ligand for IL-21R (22). The IL-21 gene consists of five exons spanning ~8.44 kb genomic DNA and is located on human chromosome 4q26-27, which is close to IL-2, a region known to be a common risk locus for multiple autoimmune diseases (35,36). IL-21 activity is mediated via binding to a compound receptor consisting of IL-21R and γ_c_, and exerts the corresponding biological effects primarily through the Janus-activated kinase/signal transducers and activators of transcription pathway or the phosphatidylinositol 3-kinase/mitogen-activated protein kinase pathway (37,38). The signaling pathways can promote T cell activation and memory, stimulate B cell differentiation and antibody production, as well as enhance the differentiation and activation of NK and Th17 cells (15,39). Thus, IL-21 may be regarded as a key cytokine linking innate and adaptive immune responses. Numerous polymorphisms of IL-21 have been identified, and it has been reported that they may directly affect the abundance of IL-21, thus, contribute to diseases (14,40). In mouse models of SLE and diabetes, increased IL-21 production was shown to be associated with autoimmunity (24,41). Furthermore, IL-21 was detected at high levels in the gut of patients with Crohn’s disease and ulcerative colitis (42,43). These observations indicate that IL-21 may be involved in the progression of multiple autoimmune diseases.

Previous study found that GD patients have markedly elevated serum levels of IL-21 compared with healthy controls (31), indicating that IL-21 may have a role in the pathogenesis of GD. In the present study, the association between polymorphisms of IL-21/rs907715 and GD was analyzed in a Southern Chinese population. The G allele of rs907715 was demonstrated to be significantly associated with an increased risk of GD development. With regard to genotype distribution analysis, the GG genotype frequencies of rs907715 in the case and control subjects were 35.1 and 23.0%, respectively (P=6.7×10^−3^). Thus, the GG genotype was significantly higher in the GD group. Similarly, the G allele of rs907715 was markedly increased in the GD cohorts, and the OR for carrying the G allele in the GD patients was 1.807 greater than the controls. These results indicated that the GG genotype and G allele of IL-21/rs907715 increased the susceptibility to GD and may be associated with the development of GD in a Cantonese population. By contrast, the AA genotype frequency of rs907715 was significantly lower in GD patients compared with the controls. The OR for carrying the AA genotype between the patients and control subjects was 0.387 (P=4.0×10^−5^; 95% CI, 0.244–0.613). Therefore, the A allele of IL-21/rs907715 may lower the risk of suffering from GD in a Cantonese population. In order to investigate whether IL-21/rs907715 is associated with a particular clinical manifestation in GD patients, correlation analyses between genotypes/alleles and clinical characteristics were performed. Notably, the GG genotype was found to be significantly increased in the relapse group when compared with the non-relapse group. In addition, the frequencies of the GG genotype in the two groups were higher than in the controls (P=2.8×10^−6^ and P=9.5×10^−4^, respectively). The G allele was significantly more frequent in the relapse group than in non-relapse group with an OR of 2.628. Thus, the G allele at the IL-21/rs907715 locus may be a significant risk factor in the susceptibility to relapse in GD patients. However, a much larger collection of cases and controls is required to confirm this association.

The observations of the present study were consistent with other studies. According to a case-control cohort study conducted in Shanghai (Eastern China), which involved 633 GD patients and 612 healthy controls, the IL-21/rs907715 SNP is significantly associated with GD, with rs907715 G allele frequencies of 54.4 and 46.3% in the GD patients and controls, respectively (χ^2^=16.05; P=6×10^−5^) (8). These results may provide further evidence for the hypothesis that IL-21/rs907715 contributes to susceptibility to GD in a Southern Chinese population. Furthermore, a statistically significant association between IL-21/rs907715 and SLE was observed in a European-American sample set, where the rs907715 SNP had a minor allele frequency of 35% in the patients as compared with 39% in the controls (χ^2^=11.55; P=6.8×10^−4^) (27). However, a Chinese population study found no association between IL-21/rs907715 polymorphisms and SLE (44). This may be due to a smaller sample size of patients, ethnic diversity and/or other various factors, including environmental and socioeconomic factors.

In addition, several studies have reported a correlation between IL-21 gene polymorphisms and a number of diseases in disparate populations (27,30,36,44,45). An association study in a Japanese population showed that the (T)7-IL-21 allele within the IL-21 gene was significantly more frequent in patients with type 1 diabetes than in control subjects (20.4 vs. 13.6%; P=0.03), indicating that the allele may be positively associated with type 1 diabetes and possible involved in the IL-21 pathway in the pathogenesis of the disease (30). Furthermore, a previous study identified that three SNPs from chromosome 4q27, containing genes for IL-2 and IL-21, were involved in the genetic susceptibility to psoriasis and psoriatic arthritis. The most significant of these was rs13151961, and the frequency of the associated T allele was significantly higher in the cases than in the controls (P=0.003) (45). Increasing evidence is supporting the role of IL-21 as a susceptibility gene contributing to multiple autoimmune diseases, including GD. However, the specific mechanisms involved require further investigation.

The results of the present study indicated that rs907715 in intron 3 of the IL-21 gene correlates with GD susceptibility. Individuals carrying risk-associated G alleles tended to be much more susceptible to GD. Furthermore, in GD patients, the G allele carriers were more susceptible to relapse, which may provide further support for the hypothesis that the G allele of IL-21/rs907715 is a risk factor of GD in a Cantonese population. However, the biological roles that the G allele may play remain poorly understood. The intronic SNP may not be the actual risk mutation, but is likely to be a surrogate marker for a mutation with functional consequences. It is possible that the associated SNP may be in linkage disequilibrium with a variant correlated with the translation of mRNA, thus, contribute to the change of protein expression or autoantibody production or the other immunological derangements in GD. However, more detailed studies are required to confirm this hypothesis.

The results of the present study provide new evidence for the presence of GD susceptibility loci in the IL-21 gene among a Southern Chinese population. Different autoimmune diseases may share similar susceptibility alleles and genetic etiologies; consequently, to a certain degree, the current results may be helpful in further clarifying variants accounting for susceptibility to other autoimmune diseases in this region. However, there are several limitations to the present study. Firstly, the number of cases and controls is insufficient. Studies with a larger sample size are required to validate the results. Secondly, GD is a complex autoimmune disease associated with other genetic and environmental factors. GD is considered to be associated with a multiple network of various susceptible loci, thus, each locus may play a small role (46). Therefore, clarifying the underlying mechanisms of IL-21 SNPs involved in the genetic predisposition to GD is necessary in further research.

In conclusion, the present study indicated that rs907715 in the IL-21 gene was significantly associated with GD in a Cantonese population from Southern China. The G allele of IL-21/rs907715 demonstrated a positive effect on the susceptibility to GD and may be a risk factor for the susceptibility to relapse in GD patients. In addition, patients carrying the A allele may have a reduced risk of suffering from GD. However, further functional studies are required to elucidate the roles in GD development at a molecular level, which may aid the identification of a potential therapeutic intervention for GD.

## Figures and Tables

**Table I:** Allele and genotype frequencies of IL-21/rs907715 in GD patients and controls.

Genotype/allele	GD, n (%)	Control, n (%)	χ^2^	P-value	OR	95% CI
Genotype						
GG	74 (35.1)	49 (23.0)	7.332^a^	6.7×10^−3^	1.797	1.173–2.753
AG	102 (48.3)	91 (43.0)	1.251^b^	0.263	1.244	0.848–1.825
AA	35 (16.6)	72 (34.0)	16.893^c^	4.0×10^−5^	0.387	0.244–0.613
Allele						
G	250 (59.2)	189 (44.6)	18.223	2.0×10^−5^	1.807	1.376–2.374
A	172 (40.8)	235 (55.4)				

Distributions of rs907715 genotypes exhibited a significant difference between the GD patients and healthy controls (χ^2^=18.500; P=9.6×10^−5^).

a To compare the frequency of the GG genotype with that of the AG and AA genotype in cases versus controls;

b to compare the frequency of the AG genotype with that of the GG and AA genotype in cases versus controls;

c to compare the frequency of the AA genotype with that of the GG and AG genotype in cases versus controls.

IL, interleukin; GD, Graves’ disease; OR, odds ratio; CI, confidence interval.

**Table II:** Correlation analyses between the genotypes and alleles of IL-21/rs907715 and the clinical characteristics of GD.

Genotype/allele	Age at onset, n (%)		Thyroid size, n (%)		Family history, n (%)		Ophthalmopathy, n (%)		Relapse history, n (%)	
				
	≤30 years	≥31 years	≤I°	≥II°	(+)	(−)	(+)	(−)	(+)	(−)
Genotype										
GG	33 (39.8)	43 (33.6)	15 (46.9)	76 (42.5)	16 (39.0)	71 (41.8)	11 (38.0)	75 (41.2)	22 (64.7)	57 (32.2)
AG	29 (34.9)	61 (47.7)	12 (37.5)	62 (34.6)	18 (43.9)	67 (39.4)	9 (31.0)	63 (34.6)	9 (26.5)	89 (50.3)
AA	21 (25.3)	24 (18.7)	5 (15.6)	41 (22.9)	7 (17.1)	32 (18.8)	9 (31.0)	44 (24.2)	3 (8.8)	31 (17.5)
χ^2^	3.453		0.846		0.280		0.628		12.866	
P-value	0.178		0.655		0.869		0.730		1.6×10^−3^	
Allele										
G	95 (57.2)	147 (57.4)	42 (65.6)	214 (59.8)	50 (61.0)	209 (61.5)	31 (53.4)	213 (58.5)	53 (77.9)	203(57.3)
A	71 (42.8)	109 (42.6)	22 (34.4)	144 (40.2)	32 (39.0)	131 (38.5)	27 (46.6)	151 (41.5)	15 (22.1)	151(42.7)
χ^2^	1.5×10^−3^		0.778		6.8×10^−3^		0.527		10.141	
P-value	0.969		0.378		0.934		0.468		1.4×10^−3^	
OR (95% CI)									2.628 (1.427–4.840)	

IL, interleukin; GD, Graves’ disease; OR, odds ratio; CI, confidence interval.

**Table III:** Allele and genotype frequencies of IL-21/rs907715 in GD patients with and without relapse history and controls.

Genotype /allele	GD, n (%)		(3) Control n (%)	χ^2^		P-value		OR (95% CI)	
			
	(1) Relapse	(2) Non-relapse		(1) vs. (3)	(2) vs. (3)	(1) vs. (3)	(2) vs. (3)	(1) vs. (3)	(2) vs. (3)
Genotype									
GG	22 (64.7)	57 (32.2)	49 (23.0)						
AG	9 (26.5)	89 (50.3)	91 (43.0)	25.588	13.910	2.8×10^−6^	9.5×10^−4^		
AA	3 (8.8)	31 (17.5)	72 (34.0)						
Allele									
G	53 (77.9)	203 (57.3)	189 (44.6)	26.103	12.583	1.0×10^−6^	3.9×10^−4^	4.393	1.672
A	15 (22.1)	151 (42.7)	235 (55.4)					(2.401–8.040)	(1.257–2.222)

IL, interleukin; GD, Graves’ disease; OR, odds ratio; CI, confidence interval.
